# Determining the cause of death through mortality surveillance using verbal autopsy in Karachi, Pakistan

**DOI:** 10.7189/jogh.15.04199

**Published:** 2025-07-21

**Authors:** Raheel Allana, Inci Yildirim, Shabina Ariff, Sameer M Belgaumi, Nazia Ahsan, Obianuju Aguolu, Sabeen Umair, Sehrish Amir Ali, Tehreem Maqsood, Mohammad Iqbal, Fauzia Aman Malik, Saad B Omer, Abdul Momin Kazi

**Affiliations:** 1 Aga Khan University, Department of Paediatrics, Karachi, Pakistan; 2 Yale School of Medicine, Department of Paediatric Infectious Diseases, New Haven, Connecticut, USA; 3 UT Southwestern Medical Centre, Peter O’ Donnell Jr School of Public Health, Dallas, Texas, USA; 4 Ohio State University, Public Health Department, Division of Epidemiology, Columbus, Ohio, USA

## Abstract

**Background:**

In Pakistan, cultural and religious beliefs restrict autopsies, limiting their prevalence. Additionally, many deaths occur at home, outside of hospital systems, making cause-of-death (CoD) determination challenging. This study aims to overcome these challenges by using a community-based verbal autopsy approach in Karachi to identify CoD.

**Methods:**

The research was conducted in two peri-urban communities within the Health Demographic Site Surveillance catchment area. A total of 1500 deaths were investigated using the World Health Organization 2016 Verbal Autopsy Questionnaire. Interviewers received extensive training to ensure culturally sensitive data collection, and physicians analysed the data to determine CoD. The 10th edition of the International Classification of Diseases (ICD-10) was integrated with verbal autopsy data for a detailed analysis of mortality causes.

**Results:**

The study identified that 52.8% of deaths were male, and 47.1% female, with 51.2% occurring in hospitals and 48.7% at home. Among home deaths, 31.5% were children under five years and 55.4% were above 18 years. Analysis revealed that major CoD included non-communicable diseases: acute cardiac disease (12.6%), liver cirrhosis (7%), and stroke (4.3%), alongside communicable diseases like diarrheal disease (6.4%), pneumonia (4.1%), and sepsis (3.4%). In adults over 18, acute cardiac disease (25.0%) and liver cirrhosis (13.1%) were prevalent, whereas neonatal sepsis (12.8%) and perinatal asphyxia (11.7%) were the most common causes in children under five years. External causes included road traffic crashes (1.6%) and accidental drowning (0.7%).

**Conclusions:**

The study underscores the need for targeted health care strategies to address the diverse CoD and varying health-seeking behaviours observed. Improving access to health care, particularly for home-based deaths and vulnerable age groups, is essential for better health outcomes. Tailored interventions are crucial to address both communicable and non-communicable diseases effectively in resource-constrained settings.

In low- and middle-income countries (LMICs), obtaining timely and precise data on the cause of death (CoD) is often a challenge due to the absence of standardised methods for collecting such information [1]. This data are vital for not only understanding the health status of a population but also for designing and evaluating public health policies and programmes. Healthcare resources are often limited in LMICs, and a clear understanding of the leading CoD can greatly assist in resource allocation and priority setting [2].

In Pakistan, like many LMICs, autopsy is often limited to medico-legal cases such as criminal deaths, suspicious or unexplained fatalities, accidents, deaths in custody, unidentified bodies, and suspected medical negligence. It is rarely performed, due to cultural beliefs, religious considerations, and resource constraints [3,4]. As a result, relying solely on autopsy data for understanding mortality patterns is not feasible. It is also estimated that around 61.2% (95% confidence interval (CI) = 48.3–73.1%) autopsies occur at home, outside of health care facilities, resulting in a significant proportion of unrecorded or unexamined mortality cases [5]. This presents a substantial challenge in accurately documenting mortality patterns and identifying underlying CoD, particularly in rural or remote areas with limited access to health care services [6]. To improve the availability of mortality data, there is a growing need to explore alternative methods for determining the CoD. A promising method of gathering this data in resource constrained settings like Pakistan is verbal autopsy (VA).

Verbal autopsies involve structured, detailed interviews with family members or caregivers of the deceased to gather information on the symptoms and circumstances leading up to death [7]. Data are then analysed using standardised algorithms or expert reviews to assign a probable CoD [8]. Verbal autopsy offers several advantages in contexts where autopsies are not feasible. First, it can be implemented in both health care settings and at the community level, allowing for a broader capture of mortality data, especially in regions where most deaths occur outside of medical facilities [9]. Additionally, the VA method is cost-effective, making it more accessible to LMICs where resources for health care and post-mortem examinations are limited [10]. Studies have also shown that when combined with medical certification and vital registration systems, VA can provide valuable insights into mortality patterns and help bridge the gap in mortality data [11,12].

Although VA is being utilised in Pakistan and other low- and middle-income settings, prior studies are largely restricted to specific age groups, such as under five years, or have relied predominantly on hospital-based data. This study represents one of the first large-scale, community-based implementations of VA encompassing both adult and paediatric populations during and post-pandemic. By adopting a community-centred surveillance framework, the study significantly contributes to a more comprehensive understanding of mortality patterns and enhances the accuracy of CoD attribution, particularly in regions with limited health care access and where conventional mortality data systems are insufficient or absent.

## METHODS

The study was conducted in two distinct peri-urban catchment areas, Ali Akbar Shah, and Bhains Colony in Karachi, Pakistan. The study was approved by the ethics review committee of the Aga Khan University (2021-6445-19025). These catchment areas encompass a culturally and ethnically diverse demographic and have robust Health and Demographic Surveillance Systems in place – providing a structured foundation for our investigation [13]. To assess cause-specific mortality through VA, we conducted interviews on a representative sample of 25% of all deaths in the two catchment areas of our study. With an estimated 6000 total deaths annually across both sites (3000 per site), this approach involved conducting VA for approximately 1500 fatalities per year (750 for each site). This sample size was expected to provide sufficient and representative data to identify leading CoD, particularly for poorly documented or misreported causes, and offer valuable insights for public health interventions. Data was collected from 2020–2022.

### Data collection

#### Training of interviewers: equipping for sensitivity and accuracy

Prior to data collection, interviewers underwent a rigorous three-day training workshop using the World Health Organization (WHO) Trainer Manual [14]. Training was facilitated by a WHO-Questionnaire master trainer and a senior social scientist and covered essential topics including data collection techniques, transcription methods, research ethics, communication skills and sensitivity training. With an understanding of the local cultural norms during the burial and bereavement period and the possible psychological stress for both participants and study staff involved in VA interviews, the interviewers were also trained to navigate this environment with care and sensitivity.

#### Death identification and VA data collection

We were notified of deaths through a WhatsApp group used for real-time reporting by demographic surveillance teams. Families were approached one week after the death occurred. This delay was necessary to give families an appropriate time to grieve – ensuring sensitivity to local cultural practices and values. Upon approach, the family was carefully briefed on the VA process. This included a comprehensive explanation of the overall purpose of the procedure – to discern the circumstances leading to the demise. A consent form detailing the VA procedure was provided to the family. Research staff ensured that family members fully understood the consent form and VA process and made clear that participation was voluntary. Consent was taken from all interviewees prior to beginning the interview. Upon consent, trained interviewers led a structured and detailed dialogue with family members or individuals close to the deceased. This comprised of a wide array of questions, probing into the signs and symptoms preceding the death, the individual's medical history, and contextual details that could shed light on potential causes. When available, interviews with the mother of the deceased were preferred. Where the mother was unavailable or deceased, the father or another close relative was approached for the VA questions. In cases where a close relative was not available during the initial approach by the team, the team would make a second visit a week later. A third visit was made in the following week if a close relative could still not be reached. The case was closed after three attempts. Each case status was noted as case closed, refused, or migrated.

#### Verbal autopsy interviews: utilising WHO 2016 Questionnaire

The WHO 2016 Verbal Autopsy Questionnaire was used to conduct interviews with next of kin or caregivers [14]. This standardised questionnaire comprises both open-ended questions for detailed symptom accounts and closed-ended questions with filter questions tailored for specific diseases. The questionnaire, presented in the local language, ensured accurate responses by facilitating a deeper understanding of the circumstances surrounding each death. To address the differing symptomatology and diagnostic challenges among age groups, separate modules within the WHO VA tool were employed for neonates (0–28 days), children (29 days–14 years), and adults (15 years and above). For neonatal deaths, interviews prioritised mothers and focused on antenatal history, birth-related events, and early-life symptoms such as poor feeding, lethargy, breathing difficulties, and signs of infection. For child deaths, interviews explored symptoms like fever, diarrhoea, seizures, cough, and signs of malnutrition, with caregivers or close relatives serving as primary respondents. Adult deaths required more detailed histories of chronic and acute symptoms, including chest pain, weight loss, jaundice, or stroke-like features. In reproductive-age women, additional questions captured obstetric history to identify potential maternal causes. All VA interviews were conducted by trained field workers using culturally sensitive approaches. To address this, preliminary meetings were held with community elders, local health workers, and religious leaders prior to study initiation. Their feedback helped refine the approach to identifying bereaved families, improve timing of consent requests, and adapt culturally appropriate language in the consent script ensuring community values were respected throughout the data collection process.

#### Determination of the CoD

Cause-of-death determination involved careful analysis of the collected data. Physicians played a crucial role in this process. Initially, one physician reviewed the case. It was then reviewed by another physician for confirmation [15]. If there was a disagreement between the first two assessments, a third physician would provide their opinion. The final CoD was determined by comparing the two main causes identified. [16]. This approach was implemented to enhance reliability and transparency in the classification of CoD. In addition, a trained psychologist accompanied the physicians during VA visits, provided grief support to parents, caregivers, or relatives of the deceased during the same visit as the VA form administration, and conducted regular counselling sessions with the study team to help them cope with the emotional toll of frequent interactions with bereaved families. This approach also fostered community trust and offered a replicable model for emotionally sensitive fieldwork.

**Figure 1:**
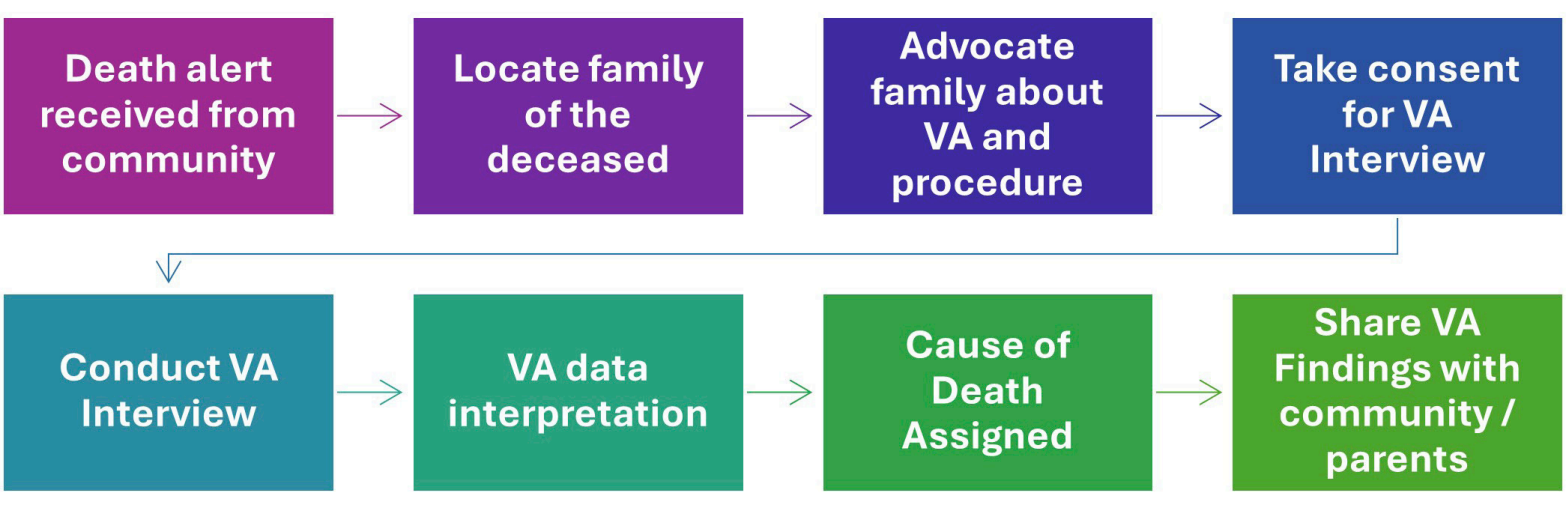
Death notification and verbal autopsy data collection process. VA – verbal autopsy.

Once the CoD was determined, the findings were shared with the community or the deceased person's family in a respectful and culturally sensitive manner. A designated member of the research team particularly the physician who has identified the CoD and someone familiar with the community, would approach the family to deliver the information. This was done in person, ensuring a private and respectful environment to allow the family time to process the information. The findings were explained in a straightforward but compassionate way, with the family given the opportunity to ask questions or seek further clarification. Throughout this process, cultural practices and privacy were prioritised by following appropriate protocols and maintaining confidentiality. The research team made sure to provide support as needed, recognising the emotional and sensitive nature of the discussion. **Figure 1** illustrates the process of VA.

### Data analysis

Trained physicians reviewed and analysed the VA questionnaires completed via interviews at the field sites. The validity of using physician review to determine CoDs for both children and adults was established, showing reasonable sensitivity and specificity for selected CoD [17]. Descriptive statistics were calculated for demographics and risk factors associated with death, providing a comprehensive overview of the patterns within the graveyard. The ICD-10 code was utilised to identify the specific CoD [15].

## RESULTS

Out of 1500 recorded deaths, 716 (47.7%) occurred in children under five years of age, while 728 (48.5%) were in adults above 18 years. Most of the deaths – around 769 (51.2%) – took place in hospitals, with 731 (48.7%) occurring at home. Ethnically, Bengalis comprised the largest group with 463 deaths (30.8%), followed by Urdu-speaking individuals with 395 (26.3%) and Sindhis with 348 deaths (23.2%) Among 785 individuals with reported education, 39.6% had no formal schooling, while among 754 with marital status data, 31.7% were married and 11.6% were widowed. (**Table 1**).

**Table 1:** Sociodemographic characteristics of deceased individuals (n = 1500)

Variable	Gender		
	**Male, n = 793 (%)**	**Female, n = 707 (%)**	**Total, n = 1500 (%)**
Age			
*<5*	384 (48.4)	332 (46.9)	716 (47.7)
*5–18*	026 (3.2)	030 (4.2)	056 (3.7)
*>18*	383 (48.3)	345 (48.8)	728 (48.5)
Place of death			
*Home*	386 (48.6)	345 (48.8)	731 (48.7)
*Hospital*	406 (51.2)	363 (51.3)	769 (51.2)
Ethnicity			
*Bengali*	268 (33.8)	195 (27.6)	463 (30.8)
*Urdu*	192 (24.2)	203 (28.7)	395 (26.3)
*Sindhi*	167 (21.0)	181 (25.6)	348 (23.2)
*Punjabi*	049 (6.2)	038 (5.3)	087 (5.8)
*Pushto*	036 (4.5)	027 (3.8)	063 (4.2)
*Hazare Wala*	014 (1.7)	011 (1.5)	015 (1.6)
*Hindko*	09 (1.1)	07 (0.9)	016 (1.0)
*Saraiki*	07 (0.8)	08 (1.1)	015 (1.0)
*Others*	26 (3.2)	021 (2.9)	047 (3.1)
Highest level of schooling (n = 785)			
*No formal education*	276 (35.1)	318 (40.5)	594 (39.6)
*Primary*	080 (10.1)	029 (3.7)	109 (7.2)
*Secondary*	035 (4.4)	022 (2.8)	057 (3.8)
*Higher secondary*	016 (2.0)	08 (1.0)	024 (1.6)
*Do not know*	01 (0.1)	-	01 (0.0)
Marital status (n = 754)			
*Married*	291 (38.6)	185 (24.5)	476 (31.7)
*Widowed*	037 (4.9)	138 (18.3)	175 (11.6)
*Single*	066 (8.7)	033 (4.3)	099 (6.6)
*Divorced*	07 (0.9)	02 (0.2)	09 (0.6)
*Refused to answer*	01 (0.13)	-	01 (0.0)

A mix of non-communicable (NCDs) and communicable diseases contributed to mortality in our population. Among NCDs, acute cardiac disease (n/N = 189/1500, 12.6%), liver cirrhosis (n/N = 105/1500, 7.0%), and stroke (n/N = 65/1500, 4.3%) were most prevalent, indicating a significant burden of chronic health conditions. Additionally, communicable diseases like diarrheal disease (n/N = 96/1500, 6.4%), pneumonia (n/N = 62/1500, 4.1%), and sepsis (n/N = 52/1500, 3.4%) were notable CoD. For children under five, neonatal causes like sepsis (n/N = 91/716, 12.7%) and perinatal asphyxia (n/N = 84/728, 11.5%) were leading contributors. In the 5–18 years category, external causes like road traffic crashes (n/N = 5/56, 8.9%) predominated, along with communicable diseases like diarrhoea and pneumonia (n/N = 4/56, 7.1%). For adults over 18 years, deaths related to NCDs were more common, such as acute cardiac disease (n/N = 182/728, 25.0%), liver cirrhosis (n/N = 96/728, 13.1%), and stroke (n/N = 64/728, 8.8%). External causes, including road traffic crashes (n/N = 15/716, 2.0%) and communicable diseases like pulmonary tuberculosis (n/N = 21/728, 2.9%), caused fewer deaths. Obstetric haemorrhage (n/N = 6/728, 0.8%) was the most common cause of maternal mortality, with pregnancy-induced hypertension and sepsis being other significant contributors. **Table 2** outlines the ICD-10 coded CoD determinations made from VA data.

**Table 2:** WHO verbal autopsy causes of death according to ICD-10 Coding (n = 1500)

WHO VA causes of death category	ICD Code	<5 y, n = 716 (%)	5–18 y, n = 56 (%)	>18 y, n = 728 (%)
**Non communicable diseases**				
Acute cardiac disease	I24	5 (0.7)	2 (3.6)	182 (25.0)
Liver cirrhosis	K74	6 (0.8)	3 (5.3)	96 (13.1)
Stroke	I64	1 (0.1)	1 (1.8)	64 (8.8)
Renal failure	N19	5 (0.7)	-	45 (6.1)
Other and unspecified disease	I99	21 (2.9)	6 (10.7)	20 (2.8)
Any other non-communicable disease	R99	8 (1.1)	3 (5.3)	18 (2.5)
Neoplasms	C06	1 (0.1)	3 (5.3)	25 (3.4)
Severe malnutrition	E46	4 (0.5)	-	1 (0.1)
Cause of death unknown	R99	1 (0.1)	-	-
Diabetes mellitus	E14	-	1 (1.8)	29 (0.3)
Respiratory neoplasms	C39	-	1 (1.8)	10 (1.4)
COPD	J44	-	-	19 (2.6)
Oral neoplasm	C26	-	-	16 (2.2)
Acute abdomen	R10	-	-	8 (1.1)
Other and unspecified neoplasms	C80	-	-	5 (0.7)
Breast neoplasms	C50	-	-	4 (0.5)
Epilepsy	G40	-	-	3 (0.3)
Asthma	J45/J46	-	-	1 (0.1)
Female reproductive neoplasms	C57	-	-	1 (0.1)
**Communicable diseases**				
Diarrhoeal disease	A09	49 (6.8)	4 (7.1)	43 (6.0)
Pneumonia	J18	42 (5.8)	4 (7.1)	14 (1.9)
Sepsis	A41	34 (4.7)	3 (5.3)	14 (1.9)
Measles	B05	36 (5.0)	3 (5.3)	-
Meningitis	G03	30 (4.1)	1 (1.8)	4 (0.5)
Other and unspecified infectious diseases	B99	5 (0.7)	2 (3.6)	7 (1.0)
Haemorrhagic fever (dengue)	A99	4 (0.5)		6 (0.8)
Pulmonary tuberculosis	A15	-	1 (1.8)	21 (2.9)
Malaria	B54	-	-	4 (0.5)
**External causes of death**				
Road traffic crashes	V89	4 (0.5)	5 (8.9)	15 (2.1)
Accidental drowning and submersion	W74	5 (0.7)	1 (1.8)	5 (0.7)
Accidental fall	W19	2 (0.3)	1 (1.8)	4 (0.5)
Other and unspecified external causes	X59	3 (0.4)	1 (1.8)	2 (0.3)
Exposure to the force of nature	X39	1 (0.1)	-	1 (0.1)
Accidental exposure to smoke, fire	X09	-	2 (3.6)	1 (0.1)
Accidental poisoning and exposure to noxious substance	X49	-	-	9 (1.2)
Unknown	R00-R99	10 (1.3)	-	14 (1.9)
**Neonatal causes of death**				
Neonatal sepsis	P63	91 (12.7)	-	-
Perinatal asphyxia	P21	84 (11.7)	-	-
Preterm birth complications	P07	52 (7.2)	-	-
Neonatal pneumonia	P23	27 (3.7)	-	-
Prematurity	P07.3	22 (3.0)	-	-
Congenital malformations	Q89	20 (2.7)	-	-
Cause not possible to determine from VA	P94	8 (1.1)	-	-
Other specific perinatal cause	P96	4 (0.5)	-	-
Term low birth weight	P07.1	3 (0.4)	-	-
Other obstetric complications	O75.4	3 (0.4)	-	-
Obstetric labour	O75	1 (0.1)	-	-
**Still births**				
Antepartum	O46.90	78 (10.8)	-	-
Intrapartum	O67.8	32 (4.4)	-	-
Other obstetric complications	O75.5	8 (1.1)	-	-
Antepartum haemorrhage	O4691	2 (0.3)	-	-
Cause not able to determine from VA	R69	2 (0.3)	-	-
Other medical conditions	R69	1 (0.1)	-	-
**Cause of maternal mortality**				
Obstetric haemorrhage (postpartum)	O72	-	-	6 (0.8)
Pregnancy-induced hypertension (pre-eclampsia)	O13	-	-	3 (0.4)
Other specific maternal causes	O05	-	-	1 (0.1)
Cause not possible to determine from VA	R69	-	-	1 (0.1)
Obstetric haemorrhage (antepartum)	O46	-	-	1 (0.1)
Other specific nonmaternal causes	O06	-	-	1 (0.1)
Pregnancy-induced hypertension (eclampsia)	O15	-	-	1 (0.1)
Pregnancy-related sepsis (antepartum)	O75.3	-	-	1 (0.1)
Unknown	R69	-	-	-
Cause of death unknown	R99	4 (0.5)	-	2 (0.3)

COPD – chronic obstructive pulmonary disease, VA – verbal autopsy

**Figure 2:**
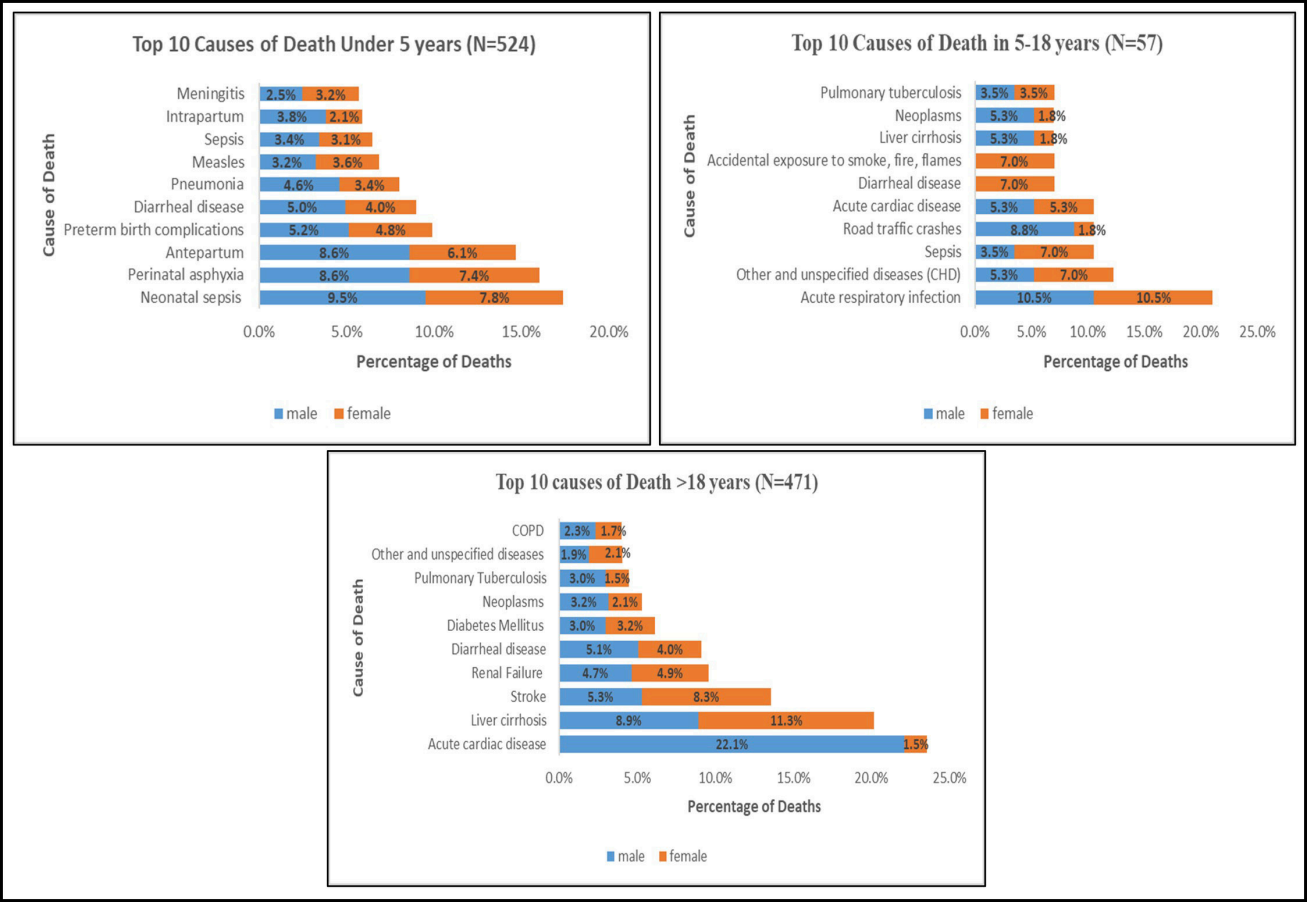
Top 10 causes of death for the three categories. CHD – congenital heart diseases, COPD – chronic obstructive pulmonary disease.

Further, among children under five years (N = 524), neonatal sepsis (9.5% males, 7.8% females), perinatal asphyxia (8.6% males, 7.4% females), and antepartum complications (8.6% males, 6.1% females) were the leading causes of death. In the 5–18 years age group (n = 57), acute respiratory infections (10.5% for both genders), road traffic crashes (8.8% males, 1.8% females), and other unspecified diseases including congenital heart diseases (CHD) (5.3% males, 7.0% females) were prominent causes. For individuals older than 18 years (n = 471), acute cardiac disease (22.1% males, 1.5% females), liver cirrhosis (8.9% males, 11.3% females), and stroke (5.3% males, 8.3% females) accounted for the highest mortality (**Figure 2**).

## DISCUSSION

The demographic insights from the VA study resonate deeply with the multifaceted nature of health care challenges in the country. Currently, the LMICs lack information regarding the specific CoD at the community level. Knowing the CoD at the population/community level is crucial since significant disparities exist between the CoD in the community and the medical setting. This study stands out as one of the few that comprehensively examined VA data for both adults and children, providing valuable insights into mortality that are essential for developing targeted public health interventions and improving health care outcomes across diverse demographic groups.

In this analysis, we have explored the distribution of death from a specific cause among adults and children and its distribution over time, sex, and age group. The present analysis showed nearly half of the death in adults were due to NCDs (41.2%), followed by deaths due to communicable diseases, neonatal causes etc. Our findings are partially consistent with analysis by the Global Burden of Disease 2019 which found ischemic heart disease, and stroke to be among the leading CoD in Pakistan [16]. In a country like Pakistan, with a population of over 200 million and approximately 190.27 million under the age of 40, there is a need to prioritise early detection, prevention, and management of NCDs, while maintaining robust surveillance and response systems for infectious diseases (Pakistan Population and Housing Census 2023).

Some LMIC studies like the Kersa health and demographic surveillance system (Kersa Health and Demographic Surveillance Systems) study of adult mortality in eastern Ethiopia, have reported lower rates of NCD associated mortality (26.4%) [18]. However, this difference may be due to differing geographical settings. Our study was conducted in an urban setting while the Kersa study population was predominantly composed of rural residents.

For a country such as Pakistan with a large percentage of the population aged under 40 years, efforts to reduce the burden of NCDs must focus on informing the youth and young adults about lifestyle choices, behaviours, and medical interventions that will reduce the risk of NCDs [16]. Although there is an aging population being increasingly affected by NCDs, investments in health systems and preventive medicine could prevent this from occurring in younger generations.

Furthermore, the leading CoD revealed in our study, such as acute cardiac disease, liver cirrhosis, diarrheal disease and pneumonia are consistent with Pakistan’s Global Burden of the Disease rates according to which ischemic heart disease and lower respiratory infections are leading CoD and contributing to a high overall mortality rate [16]. Our findings are also consistent with the Marsh DR et al. adult mortality study which examined 345 deaths between the ages of 15–59 years in a population of 45 389 in five slums in Karachi and where 55% of deaths were caused by cardiovascular diseases [19].

In our study, communicable diseases continued to remain a major cause of mortality, accounting for 23.2% of deaths. This is in line with a study conducted in Ethiopia where 30.3% of deaths were attributed to communicable diseases [20]. Other studies have also shown that the mortality burden from communicable diseases has decreased significantly over the past 30 years [21–23]. This might be due to the improvements in the health and socioeconomic status of the population over the years. In addition, a significant increase in the availability of primary health care services including vaccination could also be one factor in the observed finding.

In our study, neonatal deaths accounted for 21.2% of the total deaths, with neonatal sepsis being the predominating cause in this age group. Similarly in other LMICs, neonatal infection accounted for 17.6% of neonatal deaths [24]. The elevated rate of neonatal infections observed in this study could be attributed to inadequate health care-seeking behaviour among pregnant women. Many women seldom attend health care facilities for antenatal care, which is crucial for comprehensive maternal-foetal health management and timely detection and treatment of maternal infections. Similarly, during childbirth, many pregnant women still deliver at home and rarely seek postnatal care at health facilities [25]. This under-utilisation of health care facilities during the antenatal and peripartum periods results in inadequate access to specialised care necessary for newborns. A related factor could be the low level of female literacy in our populations. Consequently, mothers with limited education may struggle to recognise critical signs and symptoms of infections in newborns that require prompt medical attention. When symptoms are identified, many of these mothers tend to resort to local remedies for treatment [26].

Our study indicated that around one in 20 deaths (5.0%) were due to external causes with the majority being males *i.e*. 76.1%. This is in line with studies done in sub-Saharan Africa where the proportion of deaths from injury is higher among men as compared to women [27,28]. The variation in injury-related deaths between males and females can be attributed to two main factors: males often engage in activities that carry a higher risk of physical injury, and they are more frequently involved in interpersonal violence and deliberate self-harm [29–31]. In our study, the most economically active age-group (aged 18–50) are at the greatest risk of dying because of external CoD. This finding is similar to other studies in other developing countries [32]. The increased number of injury-related deaths among younger adults could suggest their heightened exposure to hazardous working conditions, such as in construction and other risky activities, compared to older adults.

Lastly, gender-specific patterns in CoD highlight nuanced health disparities. While acute cardiac disease remains a leading cause for both genders, gender-specific interventions may be needed to address certain health issues more effectively. The findings also emphasise the importance of integrated health care approaches that address both communicable and NCDs comprehensively. Strengthening primary health care systems, promoting health education and awareness, and addressing social determinants of health are crucial steps in improving health outcomes and reducing mortality rates in Pakistan.

### Strengths and limitations

The VA study provides valuable insights into mortality patterns in Pakistan, but it comes with limitations that warrant consideration. One of the main concerns is the validity of the VA methodology, as it relies on retrospective information that may be subject to recall bias or misinterpretation of symptoms by family members or caregivers. This can affect the accuracy of CoD determinations, particularly for complex medical conditions or cases with incomplete information. This is particularly relevant in cases of neonatal and maternal deaths, where the symptom descriptions provided by family members may be limited, non-specific, or absent altogether due to cultural sensitivities, limited medical knowledge, or emotional distress.

Further, incomplete data and potential sampling bias limit generalisability, and the lack of a formal power calculation restricts the ability to detect fewer common CoD. Temporal factors and evolving health care access may also affect the findings' relevance over time. Despite training, interviewer variability and subjective interpretation of responses may have introduced bias. Additionally, while physician review was used to assign CoD, inter-rater reliability metrics (*e.g*. Cohen’s kappa) were not calculated, limiting the assessment of diagnostic consistency. The exclusion of ethnicity from analytical models further constrained exploration of potential disparities.

Despite these limitations, the study also presents several strengths. One notable strength is its ability to capture data on CoD in a resource-limited setting where traditional death certification and autopsy data may be scarce. This enables the study to provide critical information about mortality patterns in areas where reliable data are often lacking. Furthermore, the use of standardised VA tools, when appropriately implemented, can improve the consistency of data collection and CoD attribution across diverse populations. The study’s findings have the potential to inform public health policies and interventions, guiding efforts to address the leading causes of mortality and improve health care delivery, particularly for vulnerable populations. Additionally, the collaboration with local health care systems and researchers enhances the study's contextual relevance and ensures that it reflects the specific health care challenges faced in Pakistan.

## CONCLUSIONS

The VA study offers valuable insights into mortality patterns and highlights the dual burden of NCDs and communicable diseases in Pakistan. These findings underscore the need for integrated strategies emphasising prevention, early detection, and stronger primary health care. While scaling up VA nationally could enhance mortality surveillance and inform health policy, its success hinges on overcoming key operational challenges such as workforce training, data standardisation, sustainable funding, and integration into existing health systems. Recognising and addressing these barriers is essential to fully realise the potential of VA as a tool for national health planning. Overall, VA remains a valuable method for informing evidence-based health care policies and interventions in Pakistan and can contribute meaningfully to improving health outcomes and well-being, particularly in under-resourced and underserved populations.

## References

[1] Hung YW Hoxha K Irwin BR Law MR Grépin KA Using routine health information data for research in low-and middle-income countries: a systematic review. BMC Health Serv Res. 2020;20:790. 10.1186/s12913-020-05660-1 32843033 PMC7446185

[2] Anderson BO Ilbawi AM El Saghir NS Breast cancer in low and middle income countries (LMIC s): a shifting tide in global health. Breast J. 2015;21:111–8. 10.1111/tbj.12357 25444441

[3] Cassum LA Refusal to autopsy: a societal practice in Pakistan context. J Clin Res Bioeth. 2014;5:198.

[4] Maixenchs M Anselmo R Zielinski-Gutiérrez E Odhiambo FO Akello C Ondire M Willingness to know the cause of death and hypothetical acceptability of the minimally invasive autopsy in six diverse African and Asian settings: a mixed methods socio-behavioural study. PLoS Med. 2016;13:e1002172. 10.1371/journal.pmed.1002172 27875532 PMC5119724

[5] Adair T Who dies where? Estimating the percentage of deaths that occur at home. BMJ Glob Health. 2021;6:e006766. 10.1136/bmjgh-2021-006766 34479953 PMC8420738

[6] Pattinson R Kerber K Waiswa P Day LT Mussell F Asiruddin S Perinatal mortality audit: counting, accountability, and overcoming challenges in scaling up in low-and middle-income countries. Int J Gynaecol Obstet. 2009;107:S113–21. 10.1016/j.ijgo.2009.07.011 19815206

[7] Nichols E Pettrone K Vickers B Gebrehiwet H Surek-Clark C Leitao J Mixed-methods analysis of select issues reported in the 2016 world health organization verbal autopsy questionnaire. PLoS One. 2022;17:e0274304. 10.1371/journal.pone.0274304 36206230 PMC9543875

[8] Quigley MA Chandramohan D Rodrigues LC Diagnostic accuracy of physician review, expert algorithms and data-derived algorithms in adult verbal autopsies. Int J Epidemiol. 1999;28:1081–7. 10.1093/ije/28.6.1081 10661651

[9] Polprasert W Rao C Adair T Pattaraarchachai J Porapakkham Y Lopez AD-of-death ascertainment for deaths that occur outside hospitals in Thailand: application of verbal autopsy methods. Popul Health Metr. 2010;8:13. 10.1186/1478-7954-8-13 20482760 PMC2881896

[10] Misganaw A Mariam DH Araya T Aneneh A Validity of verbal autopsy method to determine causes of death among adults in the urban setting of Ethiopia. BMC Med Res Methodol. 2012;12:130. 10.1186/1471-2288-12-130 22928712 PMC3568023

[11] Maduekwe NI Banjo OO Sangodapo MO Abdulazeez A Use of standard verbal autopsies to improve the mortality data capacity of civil registration and vital statistics systems in low-and middle-income countries. Demogr Res. 2023;49:219–48. 10.4054/DemRes.2023.49.10

[12] Firth SM Hart JD Reeve M Li H Mikkelsen L Sarmiento DC Integrating community-based verbal autopsy into civil registration and vital statistics: lessons learnt from five countries. BMJ Glob Health. 2021;6:e006760. 10.1136/bmjgh-2021-006760 34728477 PMC8565529

[13] Zeb MI Shahid S Naeem K Fatima U Kazi AM Jehan F Profile: maternal and child health surveillance system in Peri-urban areas of Karachi, Pakistan. Gates Open Research. 2022;2:2. 10.12688/gatesopenres.12788.2

[14] Nichols EK Byass P Chandramohan D Clark SJ Flaxman AD Jakob R The WHO 2016 verbal autopsy instrument: An international standard suitable for automated analysis by InterVA, InSilicoVA, and Tariff 2.0. PLoS Med. 2018;15:e1002486. 10.1371/journal.pmed.1002486 29320495 PMC5761828

[15] World Health Organization. The WHO application of ICD-10 to deaths during the perinatal period: ICD-PM. Geneva, Switzerland: World Health Organization; 2016.

[16] GBD 2019 Pakistan Collaborators The state of health in Pakistan and its provinces and territories, 1990–2019: a systematic analysis for the Global Burden of Disease Study 2019. Lancet Glob Health. 2023;11:e229–43. 10.1016/S2214-109X(22)00497-1 36669807 PMC10009760

[17] Leitao J Desai N Aleksandrowicz L Byass P Miasnikof P Tollman S Comparison of physician-certified verbal autopsy with computer-coded verbal autopsy for cause of death assignment in hospitalized patients in low-and middle-income countries: systematic review. BMC Med. 2014;12:22. 10.1186/1741-7015-12-22 24495312 PMC3912516

[18] Ashenafi W Eshetu F Assefa N Oljira L Dedefo M Zelalem D Trend and causes of adult mortality in Kersa health and demographic surveillance system (Kersa HDSS), eastern Ethiopia: verbal autopsy method. Popul Health Metr. 2017;15:22. 10.1186/s12963-017-0144-2 28666480 PMC5493878

[19] Marsh DR Kadir MM Husein K Luby SP Siddiqui R Khalid SB Adult mortality in slums of Karachi, Pakistan. J Pak Med Assoc. 2000;50:300–6. 11043020

[20] Fenta EH Sisay BG Gebreyesus SH Endris BS Trends and causes of adult mortality from 2007 to 2017 using verbal autopsy method, Addis Ababa, Ethiopia. BMJ Open. 2021;11:e047095. 10.1136/bmjopen-2020-047095 34785542 PMC8596056

[21] Misganaw A Mariam DH Araya T Ayele K Patterns of mortality in public and private hospitals of Addis Ababa, Ethiopia. BMC Public Health. 2012;12:1007. 10.1186/1471-2458-12-1007 23167315 PMC3520706

[22] Misganaw A Haregu TN Deribe K Tessema GA Deribew A Melaku YA National mortality burden due to communicable, non-communicable, and other diseases in Ethiopia, 1990–2015: findings from the Global Burden of Disease Study 2015. Popul Health Metr. 2017;15:29. 10.1186/s12963-017-0145-1 28736507 PMC5521057

[23] Shu J Jin W Prioritizing non-communicable diseases in the post-pandemic era based on a comprehensive analysis of the GBD 2019 from 1990 to 2019. Sci Rep. 2023;13:13325. 10.1038/s41598-023-40595-7 37587173 PMC10432467

[24] Fleischmann C Reichert F Cassini A Horner R Harder T Markwart R Global incidence and mortality of neonatal sepsis: a systematic review and meta-analysis. Arch Dis Child. 2021;106:745–52. 10.1136/archdischild-2020-320217 33483376 PMC8311109

[25] Khalid A Haider KA Ahmer H Noorani S Hoodbhoy Z Why do women still give birth at home; perceptions of Pakistani women and decision-makers from marginalized communities. PLOS Glob Public Health. 2023;3:e0002217. 10.1371/journal.pgph.0002217 37831638 PMC10575520

[26] Ahmed F Malik NI Zia S Akbar AS Li X Shahid M Rural mothers’ beliefs and practices about diagnosis, treatment, and management of children health problems: A qualitative study in marginalized Southern Pakistan. Front Public Health. 2023;10:1001668. 10.3389/fpubh.2022.1001668 36684927 PMC9845559

[27] Weldearegawi B Ashebir Y Gebeye E Gebregziabiher T Yohannes M Mussa S Emerging chronic non-communicable diseases in rural communities of Northern Ethiopia: evidence using population-based verbal autopsy method in Kilite Awlaelo surveillance site. Health Policy Plan. 2013;28:891–8. 10.1093/heapol/czs135 23293101

[28] Wolde A Abdella K Ahmed E Tsegaye F Babaniyi O Kobusingye O Pattern of injuries in Addis Ababa, Ethiopia: a one-year descriptive study. East Cent Afr J Surg. 2008;13:14–22.

[29] Chasimpha S McLean E Chihana M Kachiwanda L Koole O Tafatatha T Patterns and risk factors for deaths from external causes in rural Malawi over 10 years: a prospective population-based study. BMC Public Health. 2015;15:1036. 10.1186/s12889-015-2323-z 26449491 PMC4599750

[30] Ae-Ngibise KA Masanja H Kellerman R Owusu-Agyei S Risk factors for injury mortality in rural Tanzania: a secondary data analysis. BMJ Open. 2012;2:e001721. 10.1136/bmjopen-2012-001721 23166132 PMC3533022

[31] Mamady K Yao H Zhang X Xiang H Tan H Hu G The injury mortality burden in Guinea. BMC Public Health. 2012;12:733. 10.1186/1471-2458-12-733 22937768 PMC3489613

[32] Streatfield PK Khan WA Bhuiya A Hanifi SM Alam N Diboulo E Mortality from external causes in Africa and Asia: evidence from INDEPTH Health and Demographic Surveillance System Sites. Glob Health Action. 2014;7:25366. 10.3402/gha.v7.25366 25377327 PMC4220124

